# A phase 2 study of a combined diphtheria-tetanus-acellular pertussis vaccine with a Sabin-derived inactivated poliovirus vaccine in children

**DOI:** 10.1080/21645515.2018.1504538

**Published:** 2018-08-17

**Authors:** Takashi Nakano, Shuji Sumino, Yohei Takanami, Nodoka Mitsuya, Keisuke Nakatome

**Affiliations:** a Department of Pediatrics, Kawasaki Medical School, Kurashiki, Japan; b Takeda Pharmaceutical Company Limited, Osaka, Japan

**Keywords:** poliovirus, Sabin poliovirus, vaccine, DTaP

## Abstract

**Background**: With the goal of global eradication of poliomyelitis due to wild-type viruses within sight, WHO now recommends that infants receive at least one dose of trivalent inactivated poliovirus vaccine (IPV) with bivalent OPV (types 1 and 3) replacing trivalent OPV. Limited manufacturing capacity and new regulations on manufacturers’ use of wild-type viruses is driving the development of IPV based on attenuated Sabin type polioviruses. Takeda are developing a Sabin-based IPV (sIPV) to augment global capacity and supply.

**Methods**: This study was performed to evaluate three dosages (low, medium and high) of the sIPV when administered as a combination vaccine with diphtheria-tetanus-acellular pertussis antigens (DTaP-sIPV) as a three dose primary series or as booster dose in Japanese infants and toddlers.

**Results**: All formulations were immunogenic and well-tolerated with no safety concerns in either infants or toddlers. There was a dosage-dependent induction of neutralizing antibodies against Sabin polioviruses, the only statistically significant differences being between the low-dose and medium- and high-dose sIPVs. There was good correlation of neutralizing antibodies against Sabin and wild-type polioviruses. No sIPV dose had an observable effect on immune responses to DTaP components or the reactogenicity profile of the combined vaccine.

**Conclusion**: When administered as a DTaP-sIPV combination, Takeda’s sIPV vaccine was well-tolerated and highly immunogenic in infant and toddler schedules. The medium-dose formulation offers the optimal balance between immunogenicity and potential dose-sparing to provide a new source of sIPV to enhance the global supply, while mitigating the environmental risks associated with manufacturing vaccines with wild-type viruses.

## Introduction

Global eradication of polio disease due to infection by wild-type polioviruses is within sight, the culmination of over 60 years of immunization with live attenuated trivalent oral polio vaccines (tOPV) and trivalent inactivated injected polio vaccines (IPV). At the time of writing wild–type polio is still circulating in two countries, Pakistan and Afghanistan, the last three cases in Africa having been reported in Nigeria in July 2016.^1^ In the rest of the world the only recent cases of paralytic poliomyelitis reported are due to vaccine-associated paralytic poliomyelitis (VAPP), a rare complication occurring after 1 in 2.7 million OPV doses,^2^ or through circulating virus derived from vaccine viruses (cVDPV) originating from OPV^3^ or immunodeficiency-related vaccine-derived polioviruses (iVDPV).^4^

As disease due to wild-type 2 virus has been eradicated for over 15 years the WHO Strategic Advisory Group of Experts on Immunization (SAGE) has recommended eliminating type 2 vaccine virus by replacing tOPV with bivalent vaccine (bOPV) containing only types 1 and 3 from April 2016.^5^ To ensure some ongoing immunity against type 2 poliovirus all children should receive at least one dose of trivalent IPV, with the long term goal of eliminating the use of all forms of OPV except for emergency use in outbreaks situations.

Increased demand for IPV creates several problems. Currently, only four major manufacturers produce a limited supply of WHO prequalified IPV vaccines (Sanofi Pasteur, GlaxoSmithKline, Bilthoven Biologicals and its parent company, Serum Institute of India^6^). Expanding this capacity will be complicated by the recently introduced Global Action Plan III regulations,^6^ which increase the required containment conditions to minimize the risk of environmental release of virulent polioviruses from laboratories or IPV manufacturing facilities following recent incidents in Europe and India.^7,8^

One approach is to make IPV using the attenuated Sabin-strain polioviruses used in OPV, rather than the wild-type Salk strains used in most current IPVs outside of Japan, to eliminate the risk of virulent viruses escaping from manufacturing facilities.^9^ Takeda have agreed a technology transfer from the BIKEN Foundation (formerly the Japan Poliomyelitis Research Institute) for viral seeds to develop a Sabin IPV (sIPV), while simultaneously applying novel manufacturing technologies to enhance production capacity. This report describes the first use of Takeda sIPV in three groups of Japanese children, 3–67 months of age, when administered in a dose-ranging study as a component of a diphtheria, tetanus and acellular pertussis (DTaP-sIPV) combination vaccine.

## Results

### Study population

**Figure 1.**
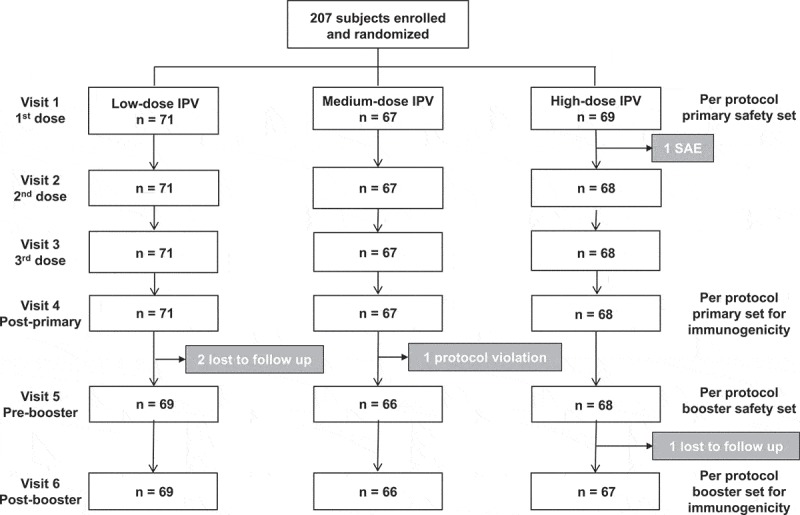
Flow chart showing numbers of subjects per group throughout the study.

A total of 207 children were enrolled and randomized to the three study groups (1:1:1) for subcutaneous vaccination with DTaP-sIPV combinations containing low-, medium- and high-doses of sIPV (Figure 1). Demographics were similar in the three study groups (Table 1). All children completed the primary vaccination series, except for one in the high-dose sIPV group who experienced an afebrile convulsion 4 days after the first vaccination and was withdrawn from the study (Figure 1). Two children in the low-dose group were lost to follow up between the primary series and the booster dose, and a third with two protocol deviations (receipt of the wrong dose vaccine formulation and an excluded medication) was withdrawn from the medium-dose group without receiving a booster dose. One high-dose subject withdrew after the booster dose as the family moved out of the study area.

**Table 1.** Demographics of the study population.

		Low-dose sIPV	Medium-dose sIPV	High-dose sIPV
	N =	71	67	69
**Age** months	**Mean ± SD** (min, max)	4.07 ± 1.68 (3.0, 16.3)	4.10 ± 1.28 (3.0, 8.5)	3.96 ± 0.95 (3.0, 7.1)
**Weight** kg	**Mean ± SD** (min, max)	6.71 ± 0.88 (4.71, 8.67)	7.01 ± 1.02 (4.76, 9.80)	6.69 ± 0.79 (4.96, 8.52)
**Height** cm	**Mean ± SD** (min, max)	61.9 ± 2.8 (55, 68)	62.4 ± 3.3 (52, 71)	62.2 ± 3.1 (56, 71)
**Gender**	**Male** **Female**	37 (52%) 34 (48%)	39 (58%) 28 (42%)	41 (59%) 28 (41%)

### Immunogenicity against sabin polioviruses

**Figure 2.**
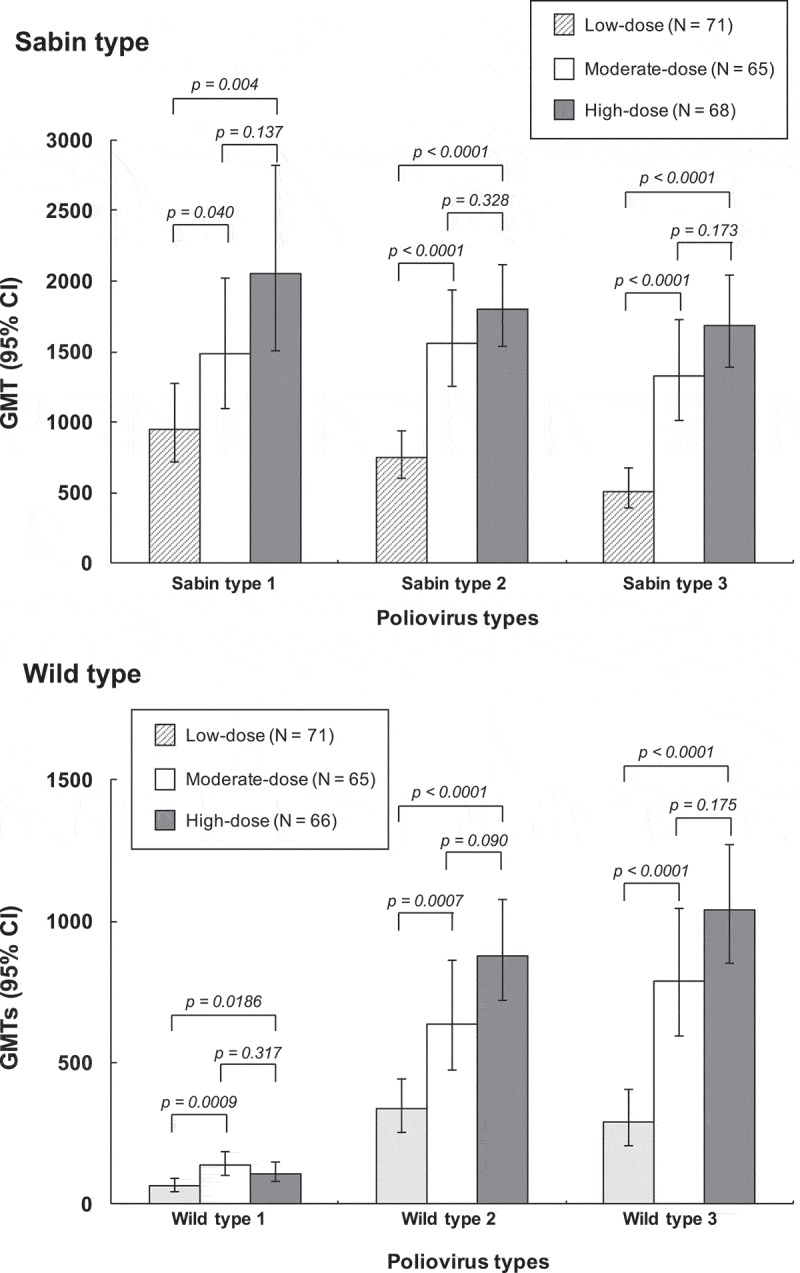
Geometric mean titers (with 95% CI) of neutralizing antibodies against the three Sabin type polioviruses (upper panel) and wild type polioviruses (lower panel) four weeks after completion of the three dose primary vaccination series.

The primary endpoint data, antibody geometric mean titers (GMTs) against the three Sabin type viruses four weeks after the primary series, are shown in Figure 2, illustrating a dosage–dependent increase in response for all three serotypes. GMTs for all serotypes were statistically significantly higher in both medium- and high-dose groups than in the low-dose group, with no significant differences between the medium- and high-dose groups.

**Figure 3.**
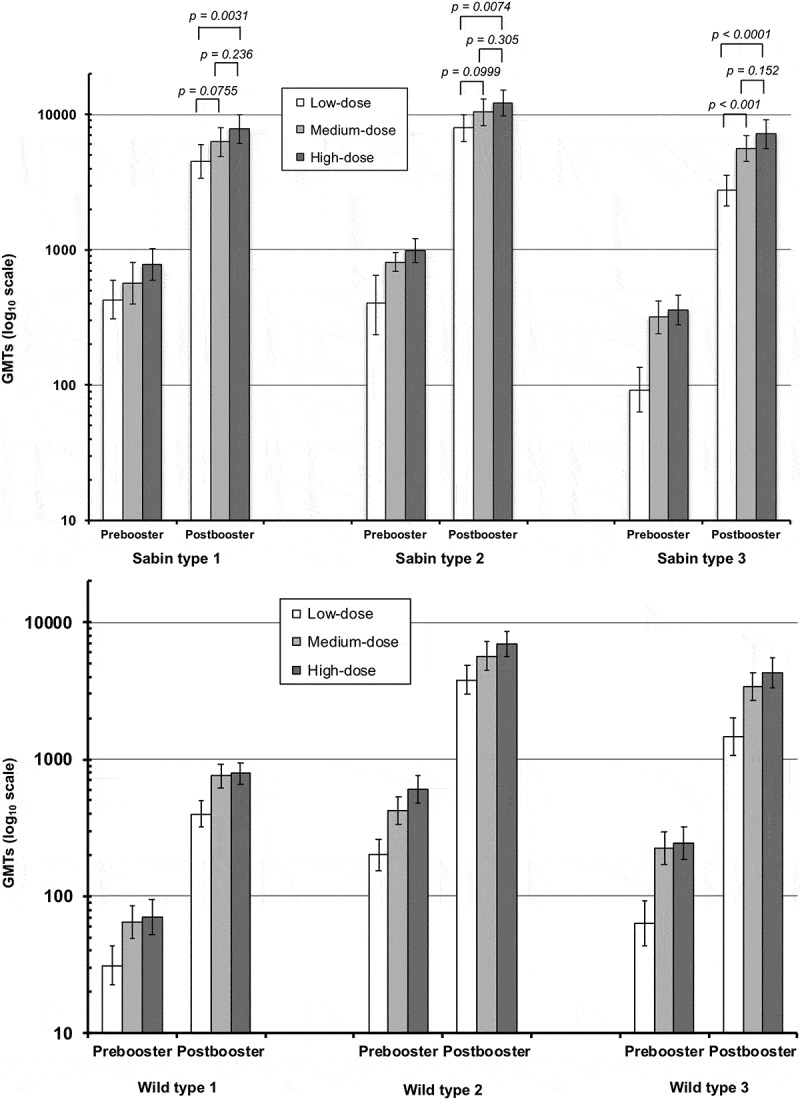
Geometric mean titers (with 95% CI, log scale) of neutralizing antibodies against the three Sabin type polioviruses (upper panel) and wild type polioviruses (lower panel) immediately before and four weeks after the booster vaccination in the three study groups.

When assessed as the seroprotection rates (SPR), the percentages (of each study group with titers ≥ 8, there were already significant proportions of children with seroprotective titers against Sabin types 1 (45.9%) and 2 (48.3%) before vaccination, presumably due to maternal antibodies (Table 2), but only 3.9% of children had protective titers against Sabin type 3. However, four weeks after completion of the primary series 100% of children in all three study groups had seroprotective titers against all three Sabin serotypes. Persistence of these antibodies and the booster effect is illustrated as GMTs in all three groups (Figure 3). The GMTs in medium- and high-dose groups were not significantly different from each other, but the high-dose group had significantly higher GMTs against all three serotypes than the low-dose group, while the medium-dose group had a significantly higher GMT than the low-dose group for Sabin type 3, but not for either type 1 or type 2 (Figure 3).

**Table 2.** Seroprotection rates (% with neutralizing titer ≥ 8) against polioviruses (FAS).

Poliovirus	Low-dose sIPV	Medium-dose sIPV	High-dose sIPV
**Primary** **Booster**	N = 71 N = 69	N = 67 N = 66	N = 69 N = 67/68
**Sabin type 1**			
Pre-vaccination	**43.7** (31.9, 56.0)	**43.3** (31.2, 56.0)	**50.7** (38.4, 63.0)
Post-primary series	**100** (94.9, 100)	**100** (94.6, 100)	**100** (94.8, 100)
Pre-booster	**98.6** (92.2, 100)	**98.5** (91.8, 100)	**100** (94.7, 100)
Post-booster	**100** (94.8, 100)	**100** (94.6, 100)	**100** (94.7, 100)
**Sabin type 2**			
Pre-vaccination	**52.1** (39.9, 64.1)	**44.8** (32.6, 57.4)	**47.8** (35.6, 60.2)
Post-primary series	**100** (94.9, 100)	**100** (94.6, 100)	**100** (94.8, 100)
Pre-booster	**100** (94.8, 100)	**100** (94.6, 100)	**100** (94.7, 100)
Post-booster	**100** (94.8, 100)	**100** (94.6, 100)	**100** (94.7, 100)
**Sabin type 3**			
Pre-vaccination	**5.6** (1.6, 13.8)	**4.5** (0.9, 12.5)	**1.4** (0.04, 7.81)
Post-primary series	**100** (94.9, 100)	**100** (94.6, 100)	**100** (94.8, 100)
Pre-booster	**92.8** (83.9, 97.6)	**100** (94.6, 100)	**100** (94.7, 100)
Post-booster	**100** (94.8, 100)	**100** (94.6, 100)	**100** (94.7, 100)
**Primary** **Booster**	N = 70/71 N = 69	N = 67/65 N = 66/65	N = 67/66 N = 67/68
Wild type 1*			
Pre-vaccination	**12.9** (6.1, 23.0)	**16.4** (8.5, 27.5)	**17.9** (9.6, 29.2)
Post-primary series	**93.0** (84.3, 97.7)	**95.4** (87.1, 99.0)	**95.5** (87.3, 99.1)
Pre-booster	**83.8** (72.9, 91.6)	**95.5** (87.3, 99.1)	**94.0** (85.4, 98.4)
Post-booster	**100** (94.7, 100)	**100** (94.5, 100)	**100** (94.7, 100)
Wild type 2*			
Pre-vaccination	**41.4** (29.8, 53.8)	**29.9** (19.3, 42.3)	**32.8** (21.8, 45.4)
Post-primary series	**100** (94.9, 100)	**100** (94.5, 100)	**100** (94.6, 100)
Pre-booster	**100** (94.7, 100)	**100** (94.6, 100)	**100** (94.6, 100)
Post-booster	**100** (94.7, 100)	**100** (94.5, 100)	**100** (94.7, 100)
Wild type 3*			
Pre-vaccination	**0** (0, 5.1)	**4.5** (0.9, 12.5)	**0** (0, 5.4)
Post-primary series	**97.2** (90.2, 99.7)	**100** (94.5, 100)	**100** (94.6, 100)
Pre-booster	**89.7** (79.9, 95.8)	**100** (94.6, 100)	**100** (94.6, 100)
Post-booster	**100** (94.7, 100)	**100** (94.5, 100)	**100** (94.7, 100)

Rates shown as percentages (95% CI)

* type 1 = Mahoney strain; type 2 = MEF-1 strain; type 3 = Sauket strain.

One year after the primary series there was some waning of the antibody titers, but GMTs remained high against all three serotypes in all three groups (Figure 3). The SPR had fallen slightly in some groups, notably the low-dose group in which 1 and 5 subjects were not seroprotected against Sabin types 1 and 3, respectively, and in the medium-dose group in which 1 subject was not protected against Sabin type 1, but four weeks after the booster dose 100% seroprotection was restored in all three groups against all three virus types (Table 2).

### Immunogenicity against wild-type polioviruses

When antibodies were measured using wild-type polioviruses results resembled those using Sabin viruses in that differences in post-primary titers were only statistically significant between the low-dose group and the other two groups, with no significant differences between medium- and high-dose groups for any serotype. After primary vaccination the SPR was 93.0–95.5% against type 1 (Mahoney strain), 100% in all three groups against type 2 (MEF-1 strain), and 97.2% in the low-dose group and 100% in the medium- and high-dose groups against type 3 (Sauket strain) (Table 2). GMTs against the three wild-type viruses four weeks after the primary vaccinations were 63–109, 336–879 and 288–1040 respectively (Figure 2). The 100% SPR was maintained against types 2 and 3 until booster, when the SPR for type 1 was 83.8–95.5% across groups. All three groups had 100% seroprotection against all three wild-type viruses after the booster dose.

There were statistically significant correlations (p < 0.0001 in all cases) between antibody titers against Sabin and wild-type polioviruses four weeks after the primary series and the booster dose for all three types in each study group. These results suggest that the antibody responses to the sIPV Sabin-type polioviruses will correlate with protection against the wild-type polioviruses in the unlikely event that the vaccinees encounter any environmental exposure to such viruses.

### Immunogenicity against dtap components

Seroprotection rates, using the defined correlates against the individual DTaP antigens, show no marked differences between the study groups (Table 3). Only 3.9% of children had protective antibodies against diphtheria toxin before vaccination, but 94.4–98.5% were protected across the three groups after the primary series. These high rates persisted until the booster vaccination, after which the SPR was 100% in all three groups. More children (57.5%) had protective anti-tetanus antibodies before vaccination, but 100% were seroprotected after the primary series, and before and after the booster in all groups.

**Table 3.** Seroprotection rates (% with indicated titers) against DTP antigens.

Antigen	Low-dose sIPV	Medium-dose sIPV	High-dose sIPV
**Primary** **Booster**	N = 71 N = 69	N = 67/66 N = 66/65	N = 69 N = 67/68
**Diphtheria** (≥ 0.1 IU/mL)			
Pre-vaccination	**5.7** (1.6, 14.0)	**4.5** (0.9, 12.5)	**1.4** (0.04, 7.8)
Post-primary series,	**94.4** (86.2, 98.4)	**98.5** (91.8, 100)	**95.7** (87.8, 99.1)
Pre-booster	**100** (94.8, 100)	**98.5** (91.8, 100)	**100** (94.6, 100)
Post-booster	**100** (94.8, 100)	**100** (94.6, 100)	**100** (94.7, 100)
**Tetanus** (≥ 0.01 IU/mL)			
Pre-vaccination	**54.9** (42.7, 66.8)	**58.2** (45.5, 70.2)	**59.4** (46.9, 71.1)
Post-primary series	**100** (94.9, 100)	**100** (94.6, 100)	**100** (94.8, 100)
Pre-booster	**100** (94.8, 100)	**100** (94.6, 100)	**100** (94.7, 100)
Post-booster	**100** (94.7, 100)	**100** (94.6, 100)	**100** (94.7, 100)
**PT** (≥ 10 EU/mL)			
Pre-vaccination	**7.0** (2.3, 15.7)	**0** (0, 5.4)	**7.2** (2.4, 16.1)
Post-primary series	**80.3** (69.1, 88.8)	**91.0** (81.5, 96.6)	**75.4** (63.5, 84.9)
Pre-booster	**14.5** (7.2, 25.0)	**15.2** (7.5, 26.1)	**10.3** (4.2, 20.1)
Post-booster	**89.9** (80.2, 95.8)	**92.4** (83.2, 97.5)	**89.7** (79.9, 95.8)
**FHA** (≥ 10 EU/mL)			
Pre-vaccination	**12.7** (6.0, 22.7)	**6.0** (1.7, 14.6)	**1.4** (0.04, 7.8)
Post-primary series	**98.6** (92.4, 100)	**100** (94.6, 100)	**100** (94.8, 100)
Pre-booster	**66.7** (54.3, 77.6)	**81.8** (70.4, 90.2)	**73.5** (61.4, 83.5)
Post-booster	**100** (94.8, 100)	**100** (94.6, 100)	**100** (94.7, 100)

There were low antibody levels against the pertussis antigens pertussis toxoid (PT) and filamentous hemagglutinin (FHA) before vaccination, with respective SPR of 4.8% and 6.8% for PT and FHA. After the primary series the PT SPR were 80.3%, 91.0% and 75.4% in low-, medium- and high-dose groups, respectively, waning to only 14.5%, 15.2% and 10.3%, respectively, before the booster, and increasing to 89.7–92.4% after the booster. FHA responses were greater in magnitude, with all children except for one in the low-dose group having protective titers, giving respective SPR of 98.6%, 100% and 100%, in the low-, medium- and high-dose groups after the primary series. Persistence of FHA was better such that before the booster 66.7–81.8% still had protective titers, which increased to 100% in all three groups after the booster dose.

### Safety

All three DTaP-sIPV formulations were generally well tolerated, as primary or booster vaccinations, unaffected by the dosage of sIPV. One serious adverse event (SAE) was considered by the investigator to be possibly related to vaccination – an afebrile convulsion in an infant in the high-dose sIPV group after the first vaccination, who was withdrawn from the study. Twenty-one infants experienced at least one SAE (5 in low-dose, 4 in medium-dose and 12 in high-dose groups) which were all SAEs were considered to be unrelated to the vaccine doses or study procedures. Most adverse events (93.0% in low-dose, 95.5% in medium-dose and 79.7% in high-dose groups) were mild, the remainder being moderate.

### Solicited local adverse events

The most frequently reported local adverse events were injection site erythema and induration, and to a lesser extent swelling. These occurred at similar rates in all three groups (Table 4), and the majority were less than 50 mm in diameter. Injection site pain was infrequent, with two or three cases in each group, and was not associated with any particular dosage of sIPV. Most local reactions occurred within 7 days of vaccination, and the majority resolved within the 14 day surveillance period, except for some cases of induration that persisted for more than 14 days. There was a trend for higher rates of local reactions after second vaccinations, with no increase after the third dose, rates were generally lower after the booster dose.

**Table 4.** Solicited local reactions and systemic adverse events reported within 14 days of each dose in the three study groups shown as numbers of cases and percentages of each group.

	Low-dose sIPV				Medium-dose sIPV				High-dose sIPV			
Dose	1st	2nd	3rd	4th	1st	2nd	3rd	4th	1st	2nd	3rd	4th
N =	71	71	71	69	67	67	67	66	69	68	68	68
**Injection site erythema**												
n (%)	30 (42)	42 (59)	37 (52)	28 (41)	25 (39)	30 (45)	29 (43)	25 (38)	28 (41)	34 (50)	27 (40)	21 (31)
**Injection site swelling**												
n (%)	11 (16)	24 (34)	16 (23)	19 (28)	6 (10)	10 (15)	11 (16)	17 (26)	4 (6)	19 (28)	15 (24)	13 (19)
**Injection site induration**												
n (%)	27 (38)	39 (55)	36 (51)	25 (36)	22 (33)	28 (42)	29 (43)	26 (39)	19 (28)	29 (43)	22 (32)	14 (21)
**Injection site pain**												
n (%)	0	1 (1.4)	1 (1.4)	0	0	2 (3.0)	0	1 (1.5)	0	0	1 (1.5)	1 (1.5)
**Pyrexia (axillary temperature)**												
≥ 37.5°C n (%)	15 (21)	7 (10)	14 (20)	18 (26)	17 (25)	12 (18)	15 (22)	19 (29)	8 (12)	15 (22)	16 (23)	19 (28)
≥ 40.0°C n (%)	-	-	-	1 (1.4)	1 (1.5)	-	-	-	-	-	-	-
**Rash**												
n (%)	2 (3)	2 (3)	0 (0)	0 (0)	3 (4)	0 (0)	1 (1)	0 (0)	5 (7)	2 (3)	1 (1)	0 (0)
**Irritability**												
n (%)	2 (3)	0 (0)	1 (1.4)	3 (4)	0 (0)	1 (1.5)	0 (0)	0 (0)	0 (0)	0 (0)	0 (0)	1 (1.5)
**Unusual Crying**												
n (%)	10 (14)	3 (4)	3 (4)	6 (6)	3 (4)	2 (3)	3 (4)	4 (6)	2 (3)	4 (6)	2 (3)	3 (4)
**Decreased appetite**												
n (%)	3 (4)	0 (0)	4 (6)	13 (19)	4 (6)	5 (7)	3 (4)	7 (11)	2 (3)	3 (4)	1 (1.4)	10 (15)
**Vomiting**												
n (%)	9 (13)	4 (6)	6 (8)	4 (6)	4 (6)	5 (7)	3 (4)	6 (9)	3 (4)	3 (4)	1 (1.4)	5 (7)
**Diarrhoea**												
n (%)	12 (17)	8 (11)	7 (10)	11 (16)	12 (18)	8 (12)	6 (9)	11 (17)	7 (10)	10 (15)	8 (12)	16 (24)
**Somnolence**												
n (%)	7 (10)	2 (3)	1 (1.4)	6 (9)	6 (9)	6 (9)	4 (6)	6 (9)	6 (9)	3 (4)	2 (3)	7 (10)
**Insomnia**												
n (%)	11 (15)	3 (4)	3 (4)	4 (6)	7 (10)	7 (10)	3 (4)	8 (12)	5 (7)	8 (12)	4 (6)	3 (4)

### Solicited systemic adverse events

The most frequently reported solicited systemic adverse events were pyrexia and diarrhea (Table 4). The rate of pyrexia did not increase with subsequent doses, and all cases resolved within the reporting period. Two cases of high fever (≥ 40°C) were reported, which resolved without sequelae. There was no association of increased systemic AEs with increasing dose levels of sIPV being combined with the licensed DTaP vaccine.

### Unsolicited adverse events

Unsolicited adverse events were general clinical manifestations that are typical in this young age group, and were equally distributed across the study arms. Most frequently reported were nasopharyngitis (in 45.1%, 44.8% and 50.7% of low-, medium- and high-dose groups, respectively), upper respiratory tract inflammation (14.1%, 29.9% and 33.3%), bronchitis (15.5%, 14.9% and 13.0%), and gastroenteritis (14.1%, 10.4% and 11.6%).

## Discussion

Globally, live oral poliovirus vaccines (OPV) will continue to be used to maintain immunity against wild-type polioviruses before their final removal when global eradication is achieved. Global eradication of wild-type 2 poliovirus has led to the WHO-led switch from trivalent OPV to bivalent OPV world-wide in April, 2016, to eliminate potential type 2 cVDPV. However, recognizing the need to maintain protection against type 2 virus, the WHO also recommends that every infant receives at least one dose of trivalent IPV in a transitional period during the post-eradication era.^5^

There is currently a global shortage of manufacturing capacity for conventional IPV using wild-type strains, with only three companies to meet the global supply needs. Furthermore, manufacturing faces increased difficulties with the new Gap III measures proposed to ensure containment of wild-type viruses,^6^ following accidental environmental contamination in India and Belgium.^7,8^ Many difficulties could be overcome using the attenuated Sabin-type viruses to prepare IPV, and it was for this reason that Takeda arranged a technology transfer of the Sabin viruses from the BIKEN Foundation.^9^ Takeda are developing a novel Sabin-based IPV vaccine, sIPV, applying innovative production methods to not only ensure containment meets Gap III requirements, but also significantly increases the yield. As most IPV in current use is being used in combination with DTP vaccines, differing dosages of sIPV were prepared for testing when administered in the form of a DTaP-sIPV combination vaccine in the present study.

As with any new vaccine intended for human use, the DTaP-sIPV combination has been subject to both preclinical animal studies and phase 1 investigations in healthy adults to ensure the general safety and tolerability. Toxicity studies in rats and monkeys detected no safety signal with the high-dose DTaP-sIPV, nor any general physiological effects (ECG, blood pressure, heart rate) in the monkey model with doses five-fold those on a weight basis to be used in humans (data not shown). A phase 1 study in eight healthy adults showed subcutaneous administration of the high-dose DTaP-sIPV to be generally well tolerated, with only mild adverse events that occurred within 2–3 days of vaccination and resolved within 7 days. No SAEs were reported, and antibodies against all components were increased such that all subjects were seropositive/seroprotected for all vaccine antigens.

This report describes the first use of Takeda’s DTaP-sIPV in the target populations of young infants and toddlers, to determine the dose level necessary to provide adequate immunity for each of the poliovirus types. Experience with wide variety of DTaP-combination vaccinations including additional antigenic components such as IPV, hepatitis B, or *Haemophilus influenzae* type b indicates that most reactions, especially in older children, are associated with the diphtheria and pertussis components. This has led to the development of combinations with lower diphtheria and pertussis antigen contents for use as booster vaccines in children, adolescents and adults.^10^ It is therefore not surprising that the different dose levels of sIPV in the present DTaP-sIPV combination formulations did not influence the reactogenicity or tolerability of these vaccines. All three DTaP-sIPV formulations were considered to be safe; although there was one related SAE (afebrile convulsion) leading to discontinuation in the high-dose sIPV group, it was not possible to attribute causality specifically to either the DTaP or the sIPV components. Indeed, occurring 4 days after vaccination it may have been a co-incidental convulsion only linked temporally to the vaccination. No other medically-significant vaccine-associated AEs were reported, and vaccines were generally well tolerated. Notably, there were no cases of the severe swelling occasionally observed with other DTaP-combination vaccines when given as boosters in toddlers and older children.^11–13^

All three sIPV dosages vaccines were immunogenic for all three serotypes. This immunogenicity significantly increased from low-to medium-dose, with no further advantage of increasing to the high-dose. Therefore use of the medium-dose might ensure some potential dose sparing resulting in more doses being manufactured. A similar pattern was observed against wild-type viruses, responses against which were correlated with those against the Sabin viruses, suggesting that immunity induced by this new sIPV component can prevent polio caused by wild type polio viruses. There was good correlations between responses to Sabin and wild-type viruses for types 2 and 3, and no difference between the medium- and high-dose groups. Lower responses against these two types with the low-dose formulation further support use of the medium-dose. Although there was less cross-reactivity against wild-type 1 than against wild-types 2 and 3 with any of the three sIPV dosages, seroprotection rates against all three wild type viruses were higher than 90% after the primary series and 100% after the booster vaccination.

There was no evidence of any clinically significant interference or effect of increasing the dose of sIPV from low- to high-dose by sIPV on the immune responses, assessed as seroprotection rates, to the components of the licensed DTaP vaccine. All three sIPV dose formulations resulted in high rates of seroprotective antibody levels against all three diseases. However, there was no control group using only DTaP, so it is not possible to analyze if there was an effect on these responses already present with the low-dose sIPV.

A similar DTaP-sIPV vaccine using bulk stocks of sIPV manufactured by the BIKEN Foundation has been developed by the Kaketsuken Research Institute for subcutaneous administration to infants and toddlers.^14^ Following a phase 2 dose-ranging study using the same dosages (in D-antigen units) as in our study, the medium-dose formulation was selected for a phase 3 study in comparison with DTaP and OPV.^14^ Our results with the novel Takeda sIPV vaccine administered subcutaneously to Japanese infants and toddlers in a combination vaccine with DTaP, are consistent with the results of the Kaketsuken DTaP-sIPV study. Interestingly, that study also found excellent cross-protection against wild-type 2 and 3 viruses, but a lower although still protective response against wild-type 1 virus as we observed with the Takeda sIPV. The most frequent adverse event to both vaccines was mild or moderate erythema. GMTs of neutralizing antibodies achieved in our study, in the range of 1000–2000 after three primary doses, are consistent with those reported in other studies of sIPV in children from Japan,^14^ China^15^ and Poland.^16^

The current development of sIPV vaccines will be a welcome addition to the armamentarium for the completion of the global eradication of poliomyelitis due to wild-type and vaccine-derived viruses. Their licensure will increase the global supply of IPV, while also facilitating increased manufacture despite the stricter GAP III requirements.^6^ Most IPV currently used is administered in the form of DTP-IPV combinations. The WHO has recently expressed a preference to continue to use whole-cell pertussis vaccine combinations (DTwP) in countries currently using DTwP, rather than switching to acellular pertussis (DTaP) vaccines, as has done in most developed countries.^17^ Therefore it is likely that this new sIPV vaccine will be of more value if used as a standalone vaccine in countries using DTwP, and further studies, including further dose-ranging studies, will be necessary to establish the immunogenicity and reactogenicity of the sIPV component when administered as a separate vaccine, either stand-alone or concomitantly administered with other routine infant vaccines including DTwP combinations in different infant populations using different schedules, particularly the EPI 6–10-14 week infant schedule.

All three studied DTaP-sIPV vaccine formulations were well-tolerated, immunogenic and suitable for use in primary series in children and as boosters in toddlers. In agreement with the previous Kaketsuken study, the dose-responses for anti-polio antibodies suggest the medium-dose sIPV is the best compromise between immunogenicity, dose-sparing and lack of interference with DTaP antigens.

Limitations of this study were the combination of sIPV with DTaP, and the schedule applied, to match that currently used in Japan. Globally, sIPV is more likely to be used as a stand-alone vaccine in the more common 6–10-14 week EPI or 2–4-6 month schedules used in other countries. An ongoing study has already been initiated in Panama to perform dose-ranging and assess the safety and immunogenicity of the stand-alone formulation in adults, toddlers and infants when administered as three doses given four weeks apart (NCT 03092791). Further licensure studies will be performed in other schedules and populations with a final selected dosage. If the results of such future studies are positive, a final Takeda sIPV formulation may be used to provide the WHO-recommended doses of IPV to infants receiving bOPV to ensure some ongoing protection against poliovirus type 2 in case of cVDPV outbreaks. In the Japanese situation assessed in this study regulators can be reassured that the responses against all three polio serotypes are high and protective.

This study confirms the safety, tolerability and immunogenicity of a novel sIPV vaccine developed from Sabin viral stocks when administered in the form of a DTaP-sIPV combination as a primary series and booster doses in Japanese infants. Further development of this sIPV vaccine as a stand-alone vaccine will be an important complement to the currently limited global supply of IPV vaccines to be used in conjunction with bOPV vaccines in the final steps of the global eradication of poliomyelitis disease.

## Methods

This phase 2, multicenter, randomized, double-blind, parallel-group study was performed in 25 sites in Japan from December 2011 to November 2013. The local IRB of each site approved the protocol and the study was performed according to local regulations and in accordance with the GCP and ICH E6 guidelines. Parents or guardians of all children provided written informed consent before enrolment, and the study was registered on the Japanese clinical trial registration site (JAPIC-CTI-111676).

### Subjects

Eligible subjects were Japanese children, 3–67 months of age, who were judged healthy based on their medical history and a physical examination at their first visit, and were available throughout the study period. Main exclusion criteria included any history of previous vaccination against any of the DTaP-sIPV components, any history of allergic reactions to vaccinations, any condition or treatment that may affect the immune response or anticipated receipt thereof (immunodeficiency, receipt of immunoglobulins or blood products or systemic corticosteroid therapy), and previous participation in any other clinical trial within 6 months of study start.

### Study design

At enrolment children were randomized to three groups (1:1:1) to receive three subcutaneous primary doses of DTaP-sIPV at four week (3–8 weeks) intervals, with a fourth dose to be given one year (6–18 months) after the third primary vaccination as a booster. The investigational vaccine consisted of constant dosages of all DTaP components which are the same as those in the licensed “Adsorbed Diphtheria-purified Pertussis-Tetanus Combined Vaccine” that was manufactured by Takeda Pharmaceutical Company Limited (15 Lf diphtheria toxoid, 2.5 Lf tetanus toxoid, ≥ 4 units pertussis toxin protective antigen per dose). To this were added three dosage levels of sIPV containing different quantities of type 1, 2 and 3 polioviruses; 0.75, 25 and 25 DU, 1.5, 50 and 50 DU and 3, 100 and 100 DU in the low-, medium- and high-dose preparations, respectively. Each 0.5 mL dose also contained 0.89 mg AlCl_3_ and 2.5 μL phenoxyethanol and was supplied in prefilled syringes to be administered by subcutaneous injection in the upper arm by blinded study personnel.

### Immunogenicity

Four 4 mL blood samples were drawn from each participant – before the first vaccination, four weeks after third primary vaccination, and before and four weeks after booster vaccination – to assess the immune responses to each vaccine component. Poliovirus antibodies were measured by *in vitro* immunoneutralization of Sabin strains (types 1, 2 and 3) and wild polio strains (type 1 [Mahoney strain], type 2 [MEF-1 strain], type 3 [Sauket strain]) with titers expressed as the reciprocal of the lowest serial dilution giving a positive result. Diphtheria toxoid antibodies were measured by immunoneutralization test using Vero cells as indicator cells, and expressed in IU/mL. Tetanus toxoid antibodies were measured by the Kaketsuken particle agglutination method, expressed in IU/mL. Anti-PT and anti-FHA antibody titers using the *Bordetella pertussis* ELISA immunoassay kit manufactured by Denka Seiken Co. Ltd (Tokyo, Japan) and expressed as EU/mL.

### Safety

Children were monitored for 30 minutes after each vaccination for immediate reactions. Parents/guardians recorded on diary cards solicited local reactions, systemic adverse events, and axillary body temperature for each of the 14 days after each vaccination, as well as any unsolicited adverse events up to 28 days. Solicited local reactions were erythema, induration, swelling (> 5 mm diameter) and pain at the injection site; solicited systemic adverse events were rash (urticarial, others), irritability, unusual crying, decreased appetite, vomiting, diarrhea, somnolence, and insomnia. In addition, 14 days after the first vaccination each child attended the clinic for assessment by the investigator. Further safety evaluations were made by telephone calls on day 15 after the following vaccinations, and all children were assessed at the clinic four weeks after each vaccination. Adverse events were monitored throughout the study period from enrolment to four weeks after the third vaccination and from the booster vaccination visit to 4 weeks later. Serious adverse events (SAE) occurring before the last study visit were to be reported immediately and followed up to resolution. The causality of adverse events to the vaccination was judged by investigator.

## Statistics

Immunogenicity analyses were performed on the full analysis set (FAS), defined as randomized subjects who received at least one vaccination. Primary study endpoints were geometric mean titers (GMTs) with two-sided 95% confidence intervals (95% CI) against polioviruses (Sabin types 1, 2 and 3) four weeks after the third vaccination. The two-sided 95% CIs were calculated by antilogarithmic conversion of the upper and lower two-sided 95% CIs of the mean of the log-transformed antibody titers. Secondary endpoints, measured at the same time-point, included seroprotection rates (SPR), defined as the proportions (%) of each study group with titers ≥ 8 against each poliovirus Sabin type, and GMTs (95% CI) and SPR against wild-type polioviruses 1, 2 and 3, and GMTs and SPR for DTaP antigens using defined protective levels (≥ 0.1 IU/mL for diphtheria toxoid, ≥ 0.01 IU/mL for tetanus toxoid, and ≥ 10 EU/mL for PT and FHA) before and after the primary series. GMTs (95% CI) and SPR for Sabin and wild-type polioviruses, and DTaP antigens were also calculated before and four weeks after booster vaccination. Descriptive comparisons of safety parameters were made in the Safety Set, defined as all subjects who received at least one vaccination of the investigational product.

Based on results previously obtained by the BIKEN foundation for responses to the three dosages of sIPV, standard deviations for the base 2 logarithms for the antibody titers to types 1, 2 and 3 were assumed to be 1.334, 1.892 and 1.939, respectively. When both the GMT ratios for the high- versus medium-dose groups, and for the medium- versus low-dose groups were assumed to be 2, the between-group differences in log base 2 transformed antibody titers would be 1. Using a 2-sample t-test for each between-group comparison at a two-sided significance level of 5%, the number of subjects required to ensure a power of 80% was 60 subjects per group. Therefore, a sample size of 198, consisting of 66 per group, was used to allow for 180 evaluable subjects after withdrawals.

## Disclosure of potential conflicts of interest

The study was financed by Takeda Pharmaceutical Company Limited including an honorarium to Dr. Takashi Nakano for study fees. Dr. Nakano has also received honoraria from Japan Vaccine Co., MSD K.K., Astellas Pharma Inc., Daiichi Sankyo Co., Mitsubishi Tanabe Pharma Corporation, Denka Seiken Co., Ltd., and Sanofi K.K. Shuji Sumino, Yohei Takanami, Nodoka Mitsuya and Keisuke Nakatome are employees of Takeda Pharmaceutical Company Limited.

## Appendix

**Table:** Incidence of serious adverse events (SAE).

Class	Reports % (n/N)		
Description	Low-dose sIPV	Medium-dose sIPV	High-dose sIPV
**Subjects with any serious treatment emergent adverse event**	7.0 (5/71)	6.0 (4/67)	18.8 (13/69)
**Cardiac disorders**	-	-	1.4 (1/69)
Tachycardia	-	-	1.4 (1/69)
**Infections & infestations**	4.2 (3/71)	4.5 (3/67)	11.6 (8/69)
Respiratory syncytial virus	1.4 (1/71)	-	2.9 (2/69)
Rotavirus	-	-	2.9 (2/69)
Atypical pneumonia	-	-	1.4 (1/69)
Bronchiolitis	-	1.5 (1/67)	-
Bronchitis	1.4 (1/71)	-	-
Bronchopneumonia	-	-	1.4 (1/69)
Gastroenteritis rotavirus	-	1.5 (1/67)	-
Influenza	-	1.5 (1/67)	-
Meningitis aseptic	1.4 (1/71)	-	-
Pneumonia	-	-	1.4 (1/69)
RSV bronchiolitis	-	-	1.4 (1/69)
RSV bronchitis	-	-	1.4 (1/69)
**Injuries & poisonings**	-	-	1.4 (1/69)
Brain contusion	-	-	1.4 (1/69)
Fall	-	-	1.4 (1/69)
Skull fracture	-	-	1.4 (1/69)
**Neoplasms (benign or malignant)**	-	-	1.4 (1/69)
Haemangioma	-	-	1.4 (1/69)
Lymphangioma	-	-	1.4 (1/69)
**Nervous system disorders**	1.4 (1/71)	1.5 (1/67)	2.9 (2/69)
Convulsion	1.4 (1/71)	1.5 (1/67)	1.4 (1/69)
Hydrocephalus	-	-	1.4 (1/69)
Subarachnoid haemorrhage	-	-	1.4 (1/69)
**Respiratory, thoracic & mediastrial disorders**	-	-	2.9 (2/69)
Pneumonia aspiration	-	-	1.4 (1/69)
Upper respiratory tract inflammation	-	-	1.4 (1/69)
**Vascular disorders**	1.4 (1/71)	-	-
Kawasaki’s disease	1.4 (1/71)	-	-
